# Moral Believer or Moral Problem-Solver? Moral Pragmatism Fosters Tolerance Without Impeding Moral Behavior

**DOI:** 10.3390/bs14110984

**Published:** 2024-10-23

**Authors:** Li Zhang, Song Tong, Kaiping Peng

**Affiliations:** 1 Department of Psychological and Cognitive Sciences, Tsinghua University, Beijing 100084, China; zhangli9099@gmail.com (L.Z.); tong.song.53w@kyoto-u.jp (S.T.); 2 Institute for Global Industry, Tsinghua University, Beijing 100084, China; 3 AI for Wellbeing Lab, Tsinghua University, Beijing 100084, China

**Keywords:** moral psychology, moral pragmatism, tolerance, multiculturalism

## Abstract

Many previous studies in moral psychology have described people as moral believers, who treat morality as universal sacred beliefs and show moral outrage and social exclusion toward people with different opinions. At the same time, moral relativism tends to make people more tolerant but also makes them question their own beliefs and leads to more immoral behavior. We propose moral pragmatism as an alternative, which treats morality as a tool for solving specific problems, thus making morality situational instead of universal, practical instead of sacred, and tolerant instead of exclusive. Through four empirical studies, we demonstrate that when moral issues are presented as practical problems rather than abstract beliefs, people consider morality to be less universal, treat dissidents with more tolerance and less outrage, and do not perform more immoral behavior at the same time. These findings highlight moral pragmatism as a flexible and culturally sensitive moral approach, promoting diverse moral perspectives and constructive cross-cultural discourses.

## 1. Introduction

The world has witnessed numerous conflicts among different groups, many of which are in the name of conflicting moral beliefs [1,2]. Many moral psychologists view these conflicts as inevitable, as in their studies, people who treat their moral beliefs as sacred and universal behaved as moral believers [3], while groups are formed by people with shared moral beliefs [1,2]. As a result, people are intolerant of those with incompatible moral beliefs. Although multicultural experience has been proposed as a solution to group conflicts caused by moral beliefs [4], studies have found that such pluralism leads to moral relativism, which impedes adherence to one’s own moral beliefs [5]. To escape this conundrum, we propose that such phenomena only arise when morality is presented in the abstract. Based on moral pragmatism [6], we suggest that people can also function as moral problem-solvers, treating morality as a practical solution to specific, real-world issues. This paper first outlines existing evidence supporting the notion of individuals as moral believers. Subsequently, we present arguments in favor of individuals as moral problem-solvers. Finally, we compare the psychological consequences of these two ways of thinking morality through four empirical studies.

### 1.1. Traditional Perspectives on Moral Beliefs

Many moral psychologists have characterized individuals as moral believers, perceiving moral beliefs to be absolute and universal truths that must be followed unconditionally [7]. For example, early studies on moral development considered the ability to universalize to be a key indicator of children’s moral development [8]. In addition, early studies have found that even small children can distinguish between local social norms and universal moral beliefs [9]. More recent studies have found that people consider moral problems to have answers that are objectively true, just like answers to problems in physics [10], while the process of universalization is found to be a key aspect of moral judgment [7]. 

Furthermore, numerous studies have found that the universality of moral beliefs is not just entertained rationally but also guarded emotionally. These beliefs are often regarded as sacred and should never be desecrated [3,11]. When others disagree with one’s core moral beliefs, individuals typically react with intense outrage [12] and distance themselves from these heretics [1,13,14]. Such staunch beliefs frequently become intertwined with one’s identity, shaping perceptions of ingroup and outgroup members [2]. Consequently, individuals who share similar moral beliefs are embraced as part of the ingroup and are often treated with favor, whereas those who diverge from these shared beliefs are ostracized as part of the outgroup.

Is it possible to respect and tolerate different moral beliefs? One proposed solution is moral relativism, which treats moral beliefs as products of acculturation. Indeed, studies have found that adopting a relativistic view of morality makes people more tolerant of other cultures’ moral beliefs and practices [4,15]. However, it can also weaken commitment to one’s own moral beliefs [5,16]. Realizing that moral beliefs vary across cultures can make one view one’s own moral beliefs as mere products of a specific culture, raising doubts about their universality and binding nature. While a universal moral belief must be followed unconditionally, a situation-bound belief leaves more room for excuses. Indeed, when primed with moral relativism, people become more “lenient” with their moral beliefs and engage in more immoral behavior [5,16]. In this sense, moral relativism exists only as the opposite side of moral absolutism, as a loss of faith in old beliefs.

### 1.2. Morality as a Problem-Solving Tool

As the studies reviewed above have shown, contemporary moral psychology often presents a binary view of human morality, presenting individuals as if they were either dogmatists or nihilists. On the one hand, people who adhere rigidly to their beliefs, unwaveringly cling to their own moral beliefs, and ostracize those who disagree can behave like moral absolutists. For instance, absolutists are more likely to hold extreme opinions and show intolerance toward political disagreement, preferring punitive measures for those who compromise [17]. On the other hand, people who adopt a nihilistic stance, viewing the world cynically, eschewing any sacrosanct values, and prioritizing self-interest, can also behave like moral relativists. For instance, individuals who read relativist arguments were more likely to engage in unethical behaviors like cheating compared to those who read absolutist arguments [16]. This dichotomy appears to trap us in a conundrum in which tolerance toward other cultures’ moral beliefs seems to demand the sacrifice of our own. 

However, we consider this to be a false dichotomy that is the result of the specific way in which morality is presented. We propose that there is at least one other way to present and discuss moral issues, that of moral pragmatism. It is inspired by the early American philosophical tradition of pragmatism championed by William James and John Dewey [6,18,19,20]. It argues that beliefs are the result of previous experiences in successfully dealing with problems in life. When the same practice works repeatedly, it becomes a habit and becomes detached from the specific conditions under which it functioned, thus a decontextualized universal belief is born out of specific contextualized practices. However, these habits are neither fixed nor rigid but amendable to new situations. That is, when faced with new situations in which old habits no longer help people overcome obstacles and reach goals, individuals will be forced to reconsider old experiences, try new ways, or learn from others to achieve their goals, thus forming new habits in the process [21,22]. This general process is applicable to all human psychology, including morality.

In this sense, from a moral pragmatist’s perspective, moral beliefs are neither universal sacred values nor mere products of culture but habits as useful tools people develop to deal with specific problems [6,23]. In familiar situations, these old habits work smoothly and help people reach their goals. Slowly but surely, they become decontextualized and are applied by default to any situation. However, when faced with new situations in which these old habits no longer work, individuals will be forced to consider other possibilities and try to learn from others [23,24,25]. For example, in James and Dewey’s time, the United States underwent industrialization and urbanization, which destroyed old institutions while creating numerous new problems, such as financial fraud, monopoly, immigration, and so on [26]. Old traditions inherited from rural communities no longer worked in the new commercialized urban settings; thus, new moral beliefs had to be created to regulate people’s behavior.

Then why do so many participants in previous studies act like moral believers? We believe the key is in how moral issues are presented. Current discussions in moral psychology often present moral issues in the abstract and refuse to provide any information on the context and consequences of moral beliefs but leave it to participants to make assumptions by themselves. For example, in Skitka’s studies [11], people proclaimed their own moral convictions and were asked how they would feel if someone disagreed with them. No reason for the other side’s belief was given. The same is true for studies on moral relativism. In Rai and Holyoak’s studies [16], participants were presented with a “weird” custom in a different culture, knowing no reason why that custom existed, nor its function in that society. These decontextualized descriptions may have dissuaded participants from appreciating different views.

From a moral pragmatist’s view, moral principles exist for a reason and serve certain functions in society, and people can be persuaded to respect other people’s values when reason is presented [15]. What is needed is a more nuanced description of the situation, that is, what problems people face and what solutions they propose. This argument also fits with Lu et al.’s findings [5], which indicated that broad but not deep multicultural experience led to increased immoral behavior, as broad multicultural experience only gave people the impression that moral beliefs are diversified but did not allow them to find out why this is so. 

It is worth noting that what we are contrasting is not abstract or concrete thinking per se, but general moral beliefs that are supposed to work in all situations and novel situations that challenge these beliefs. General moral beliefs work in most situations, and for this very reason, people hold them. As a result, people assume them to work even when information on a situation is absent, thus making them general and abstract. In addition, when people are faced with a novel situation in which an obstacle cannot be overcome by old beliefs, they need both abstract and concrete thinking to come up with a new solution. In previous studies, moral conflicts are often presented as conflicts in moral beliefs [1,2,3], which focus on the beliefs in themselves, rather than the situations in which they developed and the problems they solved. In short, our theory focuses on the situation people face, not people’s inner thoughts and beliefs. 

Our argument can be further illustrated by a comparison of the three moral narratives. The fundamental difference between the moral pragmatism perspective and the moral absolutism perspective is that according to the former perspective, there is no a priori fixed answer to moral problems. From a moral absolutism perspective, answers to moral problems are given, using either reason or emotion. From a moral pragmatism perspective, answers must be figured out in practice and are always open to further adjustments [27,28,29]. In other words, an argument from a moral absolutism perspective is supposed to have included all possible situations; thus, no new situation could surprise us, while with a moral pragmatism perspective, people are always open to new possibilities. The fundamental difference between the moral pragmatism perspective and the moral relativism perspective is that according to the former perspective, all moral beliefs must be tested in practice. Moral relativism is often presented as if any belief can be accepted through acculturation, which is absurd [28,30]. In real life, people act according to their beliefs and face the consequences. Beliefs that fail are discarded due to negative consequences. In short, from a moral pragmatism perspective, moral beliefs are held by people because they work. That is, these moral beliefs can help people overcome obstacles in the current situation and achieve their goals [31,32].

Although moral pragmatism can be traced back to early functionalist psychologists like William James and John Dewey, and while it is frequently discussed in business ethics [27,32] and educational psychology [20], it is barely mentioned in social psychology studies on moral beliefs [1,3,11,12,13]. Inspired by the moral pragmatism perspective, we intend to contrast these perspectives directly and propose that it should be possible to shape the way people think about moral issues by manipulating how moral issues are presented. While previous studies on moral absolutism and moral relativism often present moral issues in the abstract, moral pragmatism argues that moral issues should be discussed in specific contexts and as solutions to unique problems people face. As a result, if moral issues are presented as concerning abstract moral beliefs, people should behave either as moral absolutists or moral relativists; if moral issues are presented as concerning solutions to specific novel problems, people should behave as moral pragmatists. 

Specifically, because solutions are bound by specific situations, people should be less likely to consider morality to be universally applicable when moral issues are presented as solutions to problems rather than abstract beliefs. While abstract moral beliefs are a priori, existing before any specific situation is presented, and must be applied universally, practical solutions must be figured out a posterior in specific situations; thus, their applicability outside the current situation is limited. 

Furthermore, people should show less outrage and intolerance toward dissidents when moral issues are presented as solutions to problems rather than abstract beliefs. Practical solutions do not present themselves by nature and people often need to go through trials and errors to find workable solutions [33]. In this sense, different opinions should be expected on how a problem can be solved, in contrast to devoted beliefs. As a result, people should have no reason to be hostile against others proposing alternative solutions to specific problems.

Finally, since different cultures live under different circumstances and face different problems, it is natural for them to develop diverse solutions in the form of diverse moral beliefs. In addition, these culture-specific solutions pose no threat to one’s own moral beliefs because they do not claim universality. In this sense, people should be able to appreciate other people’s moral beliefs while at the same time sticking to their own moral beliefs when moral issues are presented as concrete solutions to specific problems.

### 1.3. The Present Research

In our research, we carried out four studies to determine if by presenting moral issues in different ways individuals can adopt the role of either a moral believer or moral problem-solver and consequently alter their behavior. The first study focused on whether people perceive abstract moral beliefs as more universal than concrete solutions for specific problems. The second study explored the extent to which individuals display heightened outrage and intolerance toward those who disagree with their abstract moral beliefs, as opposed to disagreements over specific problem-solving approaches. The third study repeated the first study with an added condition to deal with the mismatch in experimental materials. The fourth study involved priming participants with concepts of moral absolutism, moral relativism, and moral pragmatism to determine if moral pragmatism leads to greater tolerance for differing opinions without heightened immoral behavior. Our findings challenge the prevailing assumption that individuals are inherently moral believers. This opens the possibility for people to become more tolerant and inclusive, while at the same time remaining loyal to their own moral beliefs. 

## 2. Study 1

Many moral psychologists have traditionally posited that morality is universally held, both implicitly and explicitly, as seen in foundational research [2,8]. Universal moral beliefs are often juxtaposed with social norms that hold local relevance [9]. Despite variations in moral beliefs across cultures, individuals tend to perceive their own moral beliefs as universally applicable [34]. Research also suggests that people often view moral questions as having objectively true answers, akin to solutions to problems in physics [10]. Such as the perception of moral universalism contributes to hostility toward opposing viewpoints, predicted by the belief that if one’s moral values are universal, dissenting opinions must be erroneous. However, our perspective diverges, suggesting that this phenomenon primarily occurs when moral beliefs are presented abstractly. When addressing specific, tangible problems that cannot be solved by old experiences, people are less likely to view morality as universally applicable. Our first study tests this hypothesis.

### 2.1. Method 

Participants. This study involved a total of 200 participants, who were recruited through Credamo, URL: https://www.credamo.com/#/ (accessed on 20 May 2024), which is an online platform based in China. Participants received a nominal fee for their participation. The sample included 37 men and 163 women, with an average age of 31.77 years (*SD* = 6.78). A post hoc sensitivity power analysis revealed that a sample size of 128 participants would be sufficient to achieve 80% statistical power for detecting an effect size *d* ≥ 0.5.

Procedure. This study was a between-subject study, and participants were randomly divided into two groups: the abstract value group (AVG) and the specific problem group (SPG). Members of the AVG evaluated three moral values—birth control, polygamy, and land reform—in a randomized order, with each value presented as a declarative statement. For example, the statement for polygamy in AVG declared monogamy as the only acceptable marital form, thereby rejecting polygamy. In contrast, participants in SPG were presented with specific scenarios related to these moral values. Each scenario depicted a situation in which adhering to the corresponding moral value could conflict with resolving an urgent issue. For instance, the polygamy scenario for SPG described a country legalizing polygamy to counterbalance the significant loss of its male population due to war. Participants’ responses to the scenarios were collected post-review. Detailed descriptions of these materials are included in the Appendix A section, where the Chinese version can be found in Supplementary Materials.

Measures. The measurement phase involved participants responding to a series of structured questionnaires, which were organized as follows:

Attitude Assessment. Participants reported whether they agreed or disagreed with the presented material.

Moral Judgment. Participants reported their moral judgment with two questions (the statement/policy is correct; the statement/policy is moral) on a seven-point Likert scale, ranging from one (strongly disagree) to seven (strongly agree). The answers had high consistency (*r*s = 0.72~0.87), with statistical significance (*p*-values < 0.001).

Universalism. Participants reported their judgment of the universal applicability of the statement or policy with three questions (this statement/policy is applicable for all time; this statement/policy is applicable for all situations; this statement/policy is applicable for all cultures), using a similar seven-point Likert scale. The answers had high consistency (αs = 0.94~0.96).

Demographic Information. Finally, participants provided demographic details, specifically their genders and ages.

### 2.2. Results

This study aimed to explore participants’ perceptions of moral universalism. Consequently, we focused our analysis on those who agreed with a given moral value or solution. This approach was based on the premise that individuals who disagree with a particular moral value or solution are unlikely to consider it universal. Therefore, participants who did not align with any specific moral value or solution were excluded from subsequent analyses. 

In the context of birth control, our analysis revealed notable differences between the groups. Participants in the AVG (*N* = 63) exhibited higher moral judgment scores (5.94 ± 1.10) compared to those in the SPG (*N* = 91, 5.60 ± 0.84), as indicated by a *t*-test (*t*(152) = 2.17, *p* < 0.05, *d* = 0.35). Furthermore, AVG members also demonstrated a stronger inclination toward viewing moral principles as universal (5.06 ± 1.36) in comparison to SPG ((4.00 ± 1.82); *t*(152) = 3.90, *p* < 0.001, *d* = 0.66). 

To control the potential confounding effect of moral judgment, an analysis of covariance (ANCOVA) was conducted. In this analysis, the group served as the independent variable, moral judgment served as the covariance, and universalism served as the dependent variable. The results showed a significant main effect for the group (*F*(1, 151) = 10.20, *p* < 0.01, η^2^ = 0.06) and moral judgment (*F*(1, 151) = 49.12, *p* < 0.001, η^2^ = 0.25). Even after adjusting for moral judgment, AVG participants (adjusted *M* = 4.89, *SE* = 0.18, 95%CI [4.53, 5.25]) still viewed morality as more universal than SPG participants (adjusted *M* = 4.12, *SE* = 0.15, 95%CI [3.82, 4.42]).

For the scenario involving polygamy, results showed a similar trend. The AVG (*N* = 91, 6.27 ± 0.91) displayed a higher level of moral judgment compared to the SPG (*N* = 47, 5.24 ± 1.00), with significant differences (*t*(136) = 6.07, *p* < 0.001, *d* = 1.08). Additionally, AVG participants viewed morality as more universal (5.20 ± 1.49) than SPG participants ((3.57 ± 1.70), *t*(136) = 5.78, *p* < 0.001, *d* = 1.02). In the ANCOVA analysis, in which moral judgment was controlled as a covariate and universalism was set as the dependent variable, a significant main effect was observed for the group variable (*F*(1, 135) = 8.72, *p* < 0.01, η^2^ = 0.06). Additionally, the influence of moral judgment was found to be significant (*F*(1, 135) = 36.96, *p* < 0.001, η^2^ = 0.22). Notably, even after adjusting for moral judgment, participants in the AVG (adjusted *M* = 4.93, *SE* = 0.15, 95%CI [4.63, 5.23]) still exhibited a higher perception of moral universalism compared to those in the SPG (adjusted *M* = 4.10, *SE* = 0.22, 95%CI [3.66, 4.53]).

**Figure 1:**
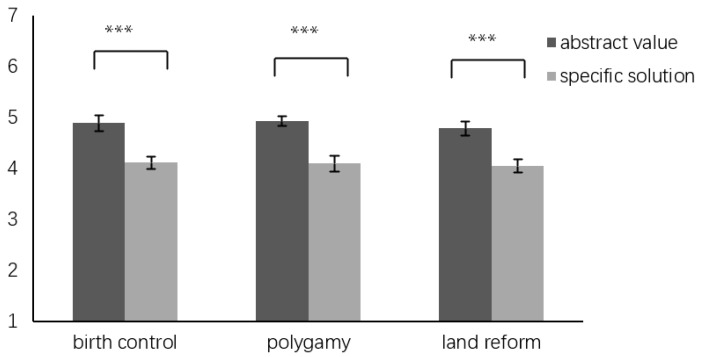
Difference in universalism. Adjusted for moral judgment. Note: *** *p* < 0.001.

In the land reform scenario, the analysis indicated comparable moral judgments between the AVG (*N* = 77, 5.77 ± 0.86) and the SPG (*N* = 98, 5.84 ± 0.78), as evidenced by a non-significant *t*-test result (*t*(173) = 0.55, *p* = 0.58, *d* = 0.09). In addition, the AVG participants viewed moral principles as more universally applicable (4.79 ± 1.57) compared to the SPG ((4.05 ± 1.71), *t*(173) = 2.95, *p* < 0.01, *d* = 0.45). These findings are summarized in Table 1 and Figure 1.

### 2.3. Discussion 

As anticipated, our study revealed that participants perceived abstract moral beliefs to be more universally applicable compared to context-specific solutions. Intriguingly, we noted that even though people may align with a particular moral belief in the abstract, they may still support a policy that starkly contradicts this belief when such a policy is useful in solving the problems at hand. It appears that people consider abstract moral beliefs and concrete moral solutions in different ways. Despite insisting that abstract moral beliefs must be universally applicable, people consider concrete solutions to real-world problems to be context-specific and are cautious to apply them to other situations.

## 3. Study 2

Previous research indicates that individuals often regard certain moral beliefs as deep convictions, reacting with intense outrage when these values are breached [12] and displaying social exclusion toward dissenters [1,13,14]. We propose the following alternative view: such intense reactions primarily emerge when moral beliefs are conceptualized abstractly. We hypothesize that when morality is framed in terms of specific, practical solutions, it is not perceived as inviolable or sacred. Consequently, contraventions of these more pragmatic moral approaches are less likely to provoke outrage or lead to social exclusion. Study 2 explores this hypothesis, experimenting with how different presentations of morality, abstract versus concrete, impact moral outrage and social tolerance toward dissenters

### 3.1. Method 

Participants. A total of 200 participants (37 male; aged 29.91 ± 6.28 years old) were recruited for this study via Credamo. They received a modest remuneration for their participation. A post hoc sensitivity power analysis showed that the minimum sample of 158 participants would provide 80% statistical power to detect an effect size *f*^2^ ≥ 0.06.

Procedure. Previous studies found that people experience outrage only when their core moral values are violated [12]. To explore this in our study, we selected the topic of polygamy, which emerged as the most widely endorsed value in Study 1, to serve as the experimental material aimed at eliciting moral outrage. This study was a between-subject study, and participants were randomly allocated to the AVG or the SPG, paralleling the methodology of Study 1. Each group was presented with materials, and participants were then asked to respond to a series of questions designed to gauge their reactions.

Measures. The participants responded to a series of questionnaires, which were organized as follows:

Attitude. Participants reported whether they agreed or disagreed with the presented material.

Moral Judgment. This was assessed using two questions on a seven-point Likert scale, ranging from one (strongly disagree) to seven (strongly agree). The questions are the same as those used in Study 1, showing high reliability (*r* = 0.85, *p* < 0.001).

Moral Outrage. Participants were informed that someone held a conflicting view on the discussed issue. Then they rated their feelings of anger, outrage, and frustration on a seven-point scale, from one (not at all) to seven (very strongly). This measure demonstrated strong internal consistency, α = 0.94.

Social Tolerance. Measured on a seven-point scale, participants expressed their willingness to engage in a social relationship with someone holding a dissenting view, such as considering them for roles like a nanny or a colleague. The scale ranged from one (very unwilling) to seven (very willing). These questions were adapted from Skitka et al. [13] and exhibited high reliability (α = 0.95).

Demographic Information. Finally, participants provided demographic details, specifically their genders and ages.

### 3.2. Results

Due to the limited number of participants who disagreed with the abstract value of monogamy (*N* = 4), we excluded them from our analyses. Subsequently, we categorized the remaining participants into three distinct groups for our study: those who agreed with the abstract value (AAV, N = 96), those who agreed with the specific solution (ASS, N = 35), and those who disagreed with the specific solution (DSS, N = 65). This categorization allowed for a more nuanced analysis of the differing perspectives within this study’s context.

Moral Judgment. A one-way ANOVA was conducted, with the group serving as the dependent variable and moral judgment serving as the independent variable. The analysis revealed a significant main effect (*F*(2, 193) = 483.21, *p* < 0.001, η^2^ = 0.83), indicating substantial differences in moral judgment across groups. The AAV group (6.28 ± 0.88, *SE* = 0.09, 95%CI [6.10, 6.45]) exhibited higher moral judgment scores compared to the ASS group (5.56 ± 0.92, *SE* = 0.16, 95%CI [5.24, 5.87], *d* = 0.80). Furthermore, both of these groups demonstrated higher moral judgment than the DSS group (2.15 ± 0.74, *SE* = 0.09, 95%CI [1.96, 2.33], *d* = 4.08).

**Figure 2:**
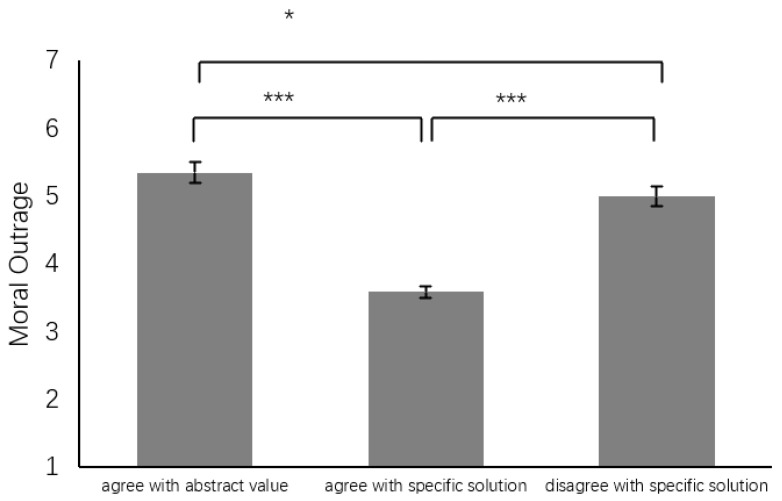
Difference in moral outrage. Note: * *p* < 0.05, *** *p* < 0.001.

Moral Outrage. A one-way ANOVA with the group as the dependent variable and moral outrage as the independent variable indicated a significant main effect (*F*(2, 193) = 19.93, *p* < 0.001, η^2^ = 0.17). The AVV group (5.35 ± 1.36, *SE* = 0.15, 95%CI [5.07, 5.64]) displayed greater moral outrage compared to the DSS group (5.00 ± 1.34, *SE* = 0.18, 95%CI [4.65, 5.34], *d* = 0.26), while the DSS group displayed greater moral outrage than the ASS group (3.59 ± 1.71, *SE* = 0.24, 95%CI [3.11, 4.06], *d* = 0.92) (see Figure 2). To assess if moral judgment influenced this outcome, an ANCOVA was executed. The significant main effect of the group remained (*F*(1, 192) = 19.99, *p* < 0.001, η^2^ = 0.17), while moral judgment did not significantly affect the result (*F*(1, 192) = 0.62, *p* = 0.43, η^2^ = 0.00). 

**Figure 3:**
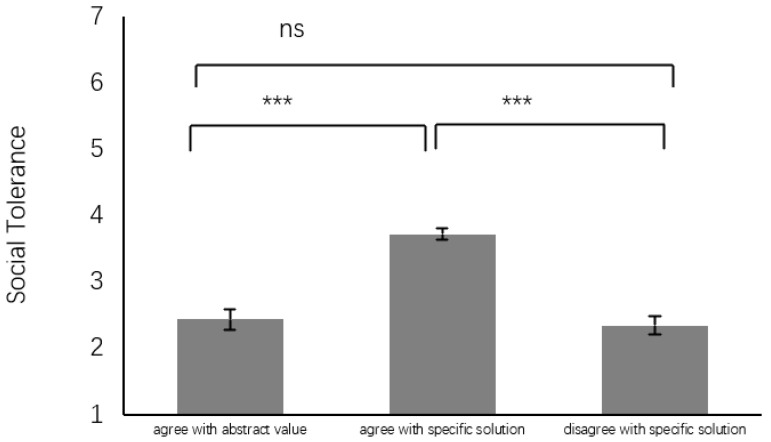
Difference in social tolerance. Note: ns: not significant, *** *p* < 0.001.

Social Tolerance. Similarly, a one-way ANOVA was performed, with social tolerance as the independent variable, revealing a significant main effect for group, (*F*(2, 193) = 18.42, *p* < 0.001, η^2^ = 0.16). The ASS group (3.72 ± 1.43, *SE* = 0.20, 95% CI [3.30, 4.11]) exhibited higher levels of social tolerance compared to both the AAV group (2.43 ± 1.18, *SE* = 0.12, 95%CI [2.19, 2.67], *d* = 0.98) and the DSS group (2.34 ± 1.01, *SE* = 0.15, 95% CI [2.05, 2.63], *d* = 1.11), with no significant difference between the latter two groups (*d* = 0.08) (see Figure 3). An ANCOVA to control for moral judgment indicated that the group effect was significant (*F*(1, 192) = 19.81, *p* < 0.001, η^2^ = 0.17), but moral judgment was not a significant factor (*F*(1, 192) = 3.04, *p* = 0.08, η^2^ = 0.02), confirming that moral judgment did not notably impact social tolerance. These findings are summarized in Table 2 and Figure 2 and Figure 3.

### 3.3. Discussion

Our findings partially substantiate our initial hypothesis. When comparing individuals who align with abstract moral values and those who endorse specific solutions, the latter group exhibited less moral outrage and greater social tolerance toward those holding dissenting opinions. However, contrary to our expectations, participants who disagreed with the specific solution displayed similar levels of moral outrage and social tolerance as those agreeing with abstract values. This outcome could be attributed to the particularly polarizing nature of the scenarios used in our study. It is plausible that those dissenting from the specific solution perceived it as excessively radical or perhaps believed there were other, more viable alternatives. Interestingly, about a third of our participants accepted the extreme solution, while concurrently demonstrating tolerance toward those who disagreed, a response pattern that was conspicuously absent when moral issues were framed in terms of abstract value.

## 4. Study 3

Study 1 made a direct comparison between people’s responses to general moral beliefs and specific situations that required radical solutions. In this study, moral beliefs were presented as decontextualized claims, which caused a mismatch between experimental conditions. Since a more complex and detailed story itself might affect how people think about moral issues, we conducted a third study to rule out this possibility. In Study 3 we repeated Study 1 with an additional condition, in which a foreign country in the scenario sticks to general moral beliefs even when it is faced with challenging specific situations. According to our hypothesis, participants under this condition should behave in a similar way to participants judging decontextualized moral beliefs, meaning that they should consider the decisions by the foreign government as universally applicable. In addition, the challenging specific situations we presented to our participants may cause a change in attitudes that might also affect people’s judgment of universalism, so we included an item on attitudinal change, as well.

### 4.1. Method 

Participants. This study involved a total of 300 participants, who were recruited through Credamo. Participants received a nominal fee for their participation. The sample included 96 men and 204 women, with an average age of 30.62 years (*SD* = 7.72). A post hoc sensitivity power analysis showed that the minimum sample of 158 participants would provide 80% statistical power to detect an effect size *f*^2^ ≥ 0.06.

Procedure. This study was a between-subject study, and participants were randomly divided into three groups: the abstract value group (AVG), the value consistent group (VCG), and the specific problem group (SPG). Members of the AVG and SPG responded to the same materials as in Study 1. Members of the VCG responded to materials similar to those of SPG, but at the end of the scenario, the foreign government chose not to implement the radical policy and gave the moral belief presented in the AVG as reasons. Participants’ responses to the scenarios were collected post-review. Detailed descriptions of these materials are included in the Appendix A and Supplementary Materials.

Measures. The measurement phase involved participants responding to a series of structured questionnaires, which were organized as follows:

Attitude Assessment. Participants reported whether they agreed or disagreed with the presented material.

Moral Judgment. Participants reported their moral judgment with two questions (the statement/policy is correct; the statement/policy is moral) on a seven-point Likert scale, ranging from one (strongly disagree) to seven (strongly agree). The answers had high consistency (*r*s = 0.74~0.85) with statistical significance (*p*-values < 0.001).

Universalism. Participants reported their judgment of the universal applicability of the statement or policy with three questions (this statement/policy is applicable for all time; this statement/policy is applicable for all situations; this statement/policy is applicable for all cultures) using a similar seven-point Likert scale. The answers had high consistency (αs = 0.94~0.96).

Attitudinal Change. Participants reported the extent that their attitude toward the issue has changed on a seven-point scale, ranging from one (not at all) to seven (totally).

Demographic Information. Finally, participants provided demographic details, specifically their genders and ages.

### 4.2. Results

Like Study 1, individuals who disagree with a particular moral value or solution are unlikely to consider it universal. Therefore, participants who did not align with a specific moral value or solution were excluded from subsequent analyses. 

In the context of birth control, a one-way ANOVA was conducted with the group as the independent variable and moral judgment as the dependent variable and revealed notable differences among groups (*F*(2, 192) = 12.10, *p* < 0.001, η^2^ = 0.11). Participants in the AVG exhibited higher moral judgment scores (*N* = 79, 6.32 ± 0.67, *SE* = 0.09, 95%CI [6.16, 6.49]) compared to those in the VCG (*N* = 21, 5.71 ± 0.86, *SE* = 0.16, 95%CI [5.39, 6.04]) and SPG (*N* = 95, 5.80 ± 0.79, *SE* = 0.08, 95%CI [5.65, 5.95]), with no significant differences between the latter two groups. A one-way ANOVA was conducted, with the group as the independent variable and attitudinal change as the dependent variable, and revealed notable differences among groups (*F*(2, 192) = 26.46, *p* < 0.001, η^2^ = 0.22). Participants in the AVG exhibited lower attitudinal change (2.81 ± 1.74, *SE* = 0.19, 95%CI [2.44, 3.18]) compared to those in the VCG (4.52 ± 1.40, *SE* = 0.37, 95%CI [3.80, 5.24]) and the SPG (4.60 ± 1.67, *SE* = 0.17, 95%CI [4.26, 4.94]), with no significant difference between the latter two groups.

A one-way ANOVA was conducted, with the group as the independent variable and universalism as the dependent variable, and revealed notable differences among groups (*F*(2, 192) = 15.67, *p* < 0.001, η^2^ = 0.14). Participants in the AVG (5.20 ± 1.51, *SE* = 0.19, 95%CI [4.82, 5.58]) were more likely to consider the expression as universally applicable than those in the VCG (4.76 ± 1.46, *SE* = 0.37, 95%CI [4.03, 5.50]), while the latter scored higher than those in the SPG (3.76 ± 1.90, *SE* = 0.18, 95%CI [3.42, 4.11]). To control the potential confounding effect of moral judgment and attitudinal change, an ANCOVA was conducted. In this analysis, the group served as the independent variable, moral judgment and attitudinal change served as the covariance, and universalism served as the dependent variable. The results showed a significant main effect for the group (*F*(2, 190) = 10.64, *p* < 0.001, η^2^ = 0.10) and moral judgment (*F*(1, 190) = 24.72, *p* < 0.001, η^2^ = 0.12) but no significant main effect for attitudinal change (*F*(1, 190) = 2.04, *p* = 0.12, η^2^ = 0.01). After adjusting for moral judgment and attitudinal change, no significant difference on universalism was found between AVG participants (adjusted *M* = 5.06, *SE* = 0.20, 95%CI [4.66, 5.45]) and VCG participants (adjusted *M* = 4.92, *SE* = 0.36, 95%CI [4.22, 5.62]), and the two groups all viewed morality as more universal than SPG participants (adjusted *M* = 3.84, *SE* = 0.18, 95%CI [3.50, 4.19]).

For the scenario involving polygamy, the results showed a similar trend. A one-way ANOVA was conducted, with the group as the independent variable and moral judgment as the dependent variable, and revealed notable differences among groups (*F*(2, 218) = 24.08, *p* < 0.001, η^2^ = 0.18). Participants in the AVG (*N* = 98, 6.29 ± 1.10, *SE* = 0.10, 95%CI [6.09, 6.48]) and the VCG (*N* = 90, 6.16 ± 0.69, *SE* = 0.10, 95%CI [5.96, 6.36]) exhibited higher moral judgment scores compared to those in the SPG (*N* = 33, 4.97 ± 1.06, *SE* = 0.17, 95%CI [4.64, 5.30]), with no significant difference between the former two groups. A one-way ANOVA was conducted, with the group as the independent variable and attitudinal change as the dependent variable, and revealed notable differences among the groups (*F*(2, 218) = 27.86, *p* < 0.001, η^2^ = 0.20). Participants in the AVG exhibited lower attitudinal change (2.49 ± 1.61, *SE* = 0.18, 95%CI [2.13, 2.85]) compared to those in the VCG (3.60 ± 2.12, *SE* = 0.19, 95%CI [3.23, 3.97]), while those in the VCG exhibited lower attitudinal change than those in the SPG (5.09 ± 1.16, *SE* = 0.31, 95%CI [4.48, 5.70]).

A one-way ANOVA was conducted, with the group as the independent variable and universalism as the dependent variable, and revealed notable differences among groups (*F*(2, 218) = 35.50, *p* < 0.001, η^2^ = 0.25). Participants in the AVG (5.13 ± 1.43, *SE* = 0.15, 95%CI [4.84, 5.42]) had similar scores on universalism compared to those in the VCG (5.30 ± 1.31, *SE* = 0.15, 95%CI [5.00, 5.60]), while these two groups scored higher than those in the SPG (2.93 ± 1.81, *SE* = 0.25, 95%CI [2.43, 3.42]). To control the potential confounding effect of moral judgment and attitudinal change, an ANCOVA was conducted. In this analysis, the group served as the independent variable, moral judgment and attitudinal change served as the covariance, and universalism served as the dependent variable. The results showed a significant main effect for the group (*F*(2, 216) = 17.90, *p* < 0.001, η^2^ = 0.14) and moral judgment (*F*(1, 216) = 40.08, *p* < 0.001, η^2^ = 0.16) but no significant main effect for attitudinal change (*F*(1, 216) = 2.20, *p* = 0.14, η^2^ = 0.01). After adjusting for moral judgment and attitudinal change, no significant difference in universalism was found between AVG participants (adjusted *M* = 5.05, *SE* = 0.14, 95%CI [4.77, 5.33]) and VCG participants (adjusted *M* = 5.21, *SE* = 0.14, 95%CI [4.93, 5.49]), and the two groups all viewed morality as more universal than SPG participants (adjusted *M* = 3.43, *SE* = 0.26, 95%CI [2.91, 3.95]).

Similar results were also found in the land reform scenario. A one-way ANOVA was conducted, with the group as the independent variable and moral judgment as the dependent variable, and revealed notable differences among groups (*F*(2, 199) = 4.43, *p* < 0.05, η^2^ = 0.04). Participants in the AVG exhibited similar moral judgment scores (*N* = 75, 5.97 ± 0.78, *SE* = 0.09, 95%CI [5.81, 6.14]) compared to those in the SPG (*N* = 96, 6.07 ± 0.69, *SE* = 0.08, 95%CI [5.92, 6.22]), and the two groups made higher moral judgments than those in the VCG (*N* = 31, 5.61 ± 0.78, *SE* = 0.13, 95%CI [5.35, 5.88]). A one-way ANOVA was conducted, with the group as the independent variable and attitudinal change as the dependent variable, and revealed notable differences among the groups (*F*(2, 199) = 24.68, *p* < 0.001, η^2^ = 0.20). Participants in the AVG exhibited lower attitudinal change (3.04 ± 1.57, *SE* = 0.19, 95%CI [2.67, 3.41]) compared to those in the VCG (4.26 ± 1.93, *SE* = 0.29, 95%CI [3.68, 4.83]), while those in the VCG exhibited lower attitudinal change than those in the SPG (4.79 ± 1.56, *SE* = 0.17, 95%CI [4.46, 5.12]).

**Figure 4:**
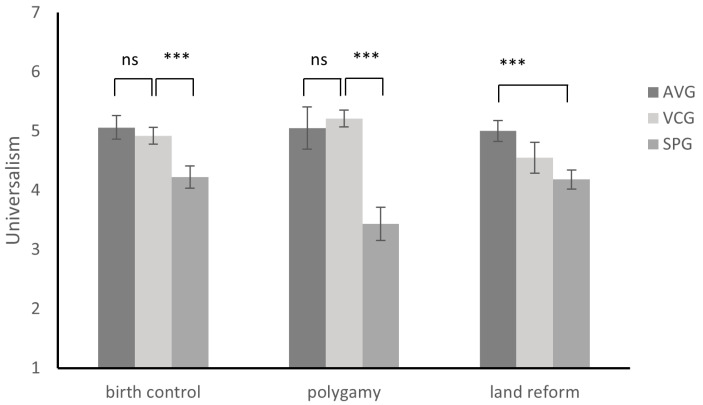
Difference on universalism. Adjusted for moral judgment and attitudinal change. Note: ns: not significant, *** *p* < 0.001.

A one-way ANOVA was conducted, with the group as the independent variable and universalism as the dependent variable, and revealed notable differences among groups (*F*(2, 199) = 2.28, *p* = 0.10, η^2^ = 0.02). Participants in the AVG (4.85 ± 1.51, *SE* = 0.19, 95%CI [4.49, 5.23]) were more likely to consider the expression as universally applicable than those in the VCG (4.31 ± 1.46, *SE* = 0.29, 95%CI [3.74, 4.89]) and the SPG (4.37 ± 1.90, *SE* = 0.17, 95%CI [4.04, 4.70]). To control the potential confounding effect of moral judgment and attitudinal change, an ANCOVA was conducted. In this analysis, the group served as the independent variable, moral judgment and attitudinal change served as the covariance, and universalism served as the dependent variable. The results showed a significant main effect for the group (*F*(2, 197) = 4.83, *p* < 0.01, η^2^ = 0.05), moral judgment (*F*(1, 197) = 26.82, *p* < 0.001, η^2^ = 0.12), and attitudinal change (*F*(1, 197) = 4.79, *p* < 0.05, η^2^ = 0.02). After adjusting for moral judgment and attitudinal change, AVG participants (adjusted *M* = 5.00, *SE* = 0.19, 95%CI [4.63, 5.37]) scored higher than SPG participants on universalism (adjusted *M* = 4.18, *SE* = 0.16, 95%CI [3.86, 4.51]), with VCG participants in the middle (adjusted *M* = 4.55, *SE* = 0.28, 95%CI [4.00, 5.10]). These findings are summarized in Table 3 and Figure 4.

### 4.3. Discussion 

Study 3 repeated the results of Study 1 in that people considered general moral beliefs to be more universally applicable than specific solutions proposed in challenging situations. Furthermore, even when these general moral beliefs were presented not as idle claims but as responses to challenging situations, people still considered them to be more universally applicable than specific solutions. This result ruled out the possibility that our findings in Study 1 were due to a mismatch between conditions. Finally, although after reading challenging specific situations, participants changed their attitudes to some extent, it did not interfere with their judgment of universal applicability. These findings were consistent with our hypotheses and strengthened our argument that general moral beliefs were more likely to be seen as universal than solutions to specific problems.

## 5. Study 4

Prior research has established that when primed with moral absolutism, people tend to be intolerant toward dissidents yet refrain from immoral behaviors, whereas when primed with moral relativism, despite tolerance for differing views, people are often inclined toward immoral behavior [16]. This dichotomy is largely attributed to multicultural exposure, which exposes the diversity of moral beliefs and leads individuals to reassess and often relax their moral standards [5]. Our study, however, posits that such a binary outcome predominantly occurs when morality is conceptualized in abstract terms. We propose that adopting a moral pragmatic approach, wherein moral beliefs are perceived as culturally developed tools for addressing specific problems, allows for both the tolerance of diverse moral viewpoints and adherence to personal standards. In addition, in Study 1 and Study 2, abstract moral beliefs were presented as idle claims, while concrete solutions were presented in detailed situations. This asymmetry might include confounding factors. To rule out this possibility and further investigate how priming affects immoral behavior, Study 3 directly contrasts the narratives of moral absolutism, moral relativism, and moral pragmatism. We hypothesize that when individuals understand the contextual reasons behind unique cultural moral principles, they become more tolerant of those differences without compromising their own moral principles, thus avoiding an increase in immoral behaviors.

### 5.1. Method 

Participants. This study engaged 150 individuals (54 male; aged 29.37 ± 6.98 years old) recruited through Credamo, who received a nominal fee for their participation. To ensure adequate statistical power, a post hoc sensitivity analysis was conducted, confirming that a minimum sample size of 100 participants would provide 80% statistical power to detect an effect size *f*^2^ ≥ 0.10.

Procedure. This study was a between-subject study, and participants were randomly allocated to one of three groups: the moral absolutism group (MAG), the moral relativism group (MRG), and the moral pragmatism group (MPG). Each group was presented with a short vignette discussing cultural variations in marriage. The MAG encountered material framing morality as absolute and universal, critically assessing various marital practices across cultures and advocating monogamy as the only ethical form due to its equal treatment of both genders. The MRG’s material positioned morality as a construct of culture and education, showcasing diverse marital customs and promoting respect for different cultural norms without imposing one’s own moral values. For the MPG, the material illustrated morality as a tool for addressing specific societal challenges, encouraging an understanding of other cultures’ moral values in the context of their unique situation. After the vignettes, participants responded to questions derived and adapted from Rai and Holyoak’s previous research [16], which was focused on these perspectives.

Measures. The measurement phase involved participants responding to a series of structured questionnaires, which were organized as follows:

Attitude. Participants reported whether they agreed or disagreed with the presented material.

Persuasiveness. Participants evaluated the persuasiveness of the material using a seven-point scale, where one represented “not at all persuaded” and seven indicated “totally persuaded”. 

Attitude Change. Participants were asked to assess the extent to which their views on monogamy shifted after engaging with the material. This was measured on a seven-point scale, ranging from one (not at all) to seven (totally).

Tolerance. Participants responded to a scenario involving polygamy’s occasional acceptability, evaluating their willingness to accept such an individual as a roommate. This assessment utilized a seven-point scale, with one indicating absolutely unacceptability and seven representing complete acceptability. Here we stick to the measures used by Rai and Holyoak [16], which are significantly shorter, rather than the one used in Study 2 to prevent the priming effect from fading away.

Immoral Behavior. Participants were presented with a hypothetical scenario adopted from Rai and Holyoak [16] involving an opportunity to purchase a mispriced product at a significantly lower cost through an automated checkout. They were asked to rate, on a seven-point scale, the likelihood of engaging in this action, with one signifying absolute refusal and seven indicating definite willingness. This measure aimed to assess participants’ propensity for immoral behavior in a situation presenting ethical ambiguity and personal gain. 

### 5.2. Results

Persuasiveness and Attitude Change. A one-way between-subjects ANOVA was conducted to evaluate the persuasiveness of each moral perspective, showing a significant group effect (*F*(2, 147) = 5.66, *p* < 0.01, η^2^ = 0.08). A post hoc analysis revealed that the MRG’s persuasiveness (4.64 ± 1.76, *SE* = 0.22, 95%CI [4.21, 5.07]) was significantly lower than that of the MPG (5.10 ± 1.49, *SE* = 0.22, 95%CI [4.67, 5.53]), while the latter’s persuasiveness was significantly lower than that of the MAG (5.68 ± 1.38, *SE* = 0.22, 95%CI [5.25, 6.11]). Another ANOVA focusing on attitude change revealed no significant main effect (*F*(2, 147) = 0.45, *p* = 0.64, η^2^ = 0.01), suggesting that the materials did not significantly alter participants’ views on monogamy. These results align with previous findings by Rai and Holyoak (2013) [16].

Tolerance. To assess tolerance levels, a one-way ANOVA was conducted, with tolerance as the criterion. This analysis reveals a significant difference among groups (*F*(2, 147) = 13.21, *p* < 0.001, η^2^ = 0.15). A subsequent post hoc analysis indicated that the MRG (3.96 ± 1.99, *SE* = 0.25, 95%CI [3.47, 4.45]) is significantly higher than the MPG (3.10 ± 1.87, *SE* = 0.25, 95%CI [2.61, 3.59]), while the latter is significantly higher than the MAG (2.16 ± 1.33, *SE* = 0.25, 95%CI [1.67, 2.65]).

**Figure 5:**
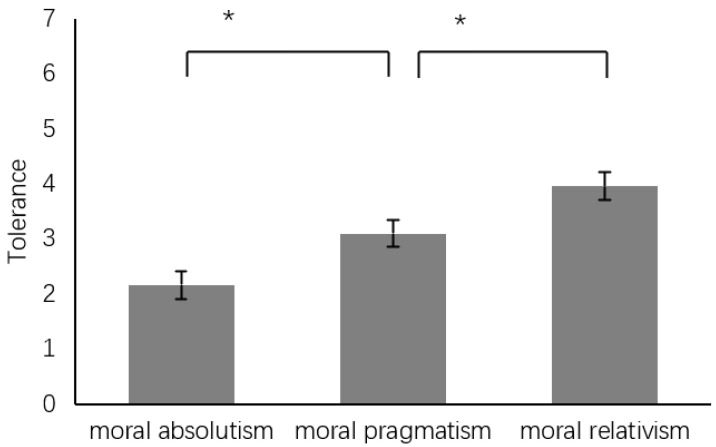
Difference in tolerance. Note: * *p* < 0.05.

**Figure 6:**
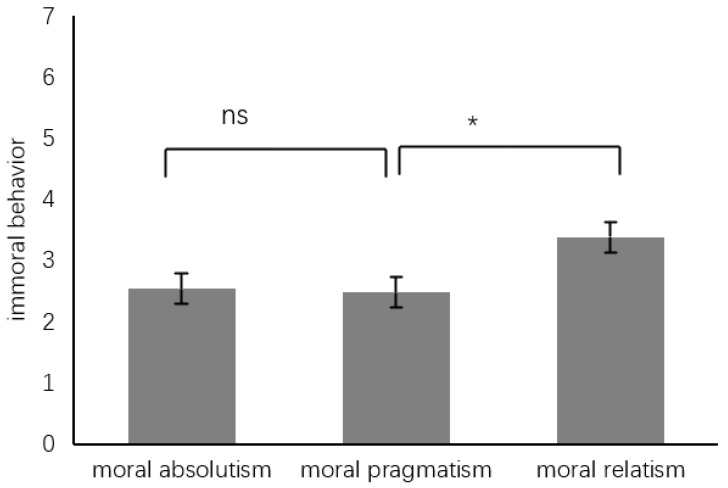
Difference in immoral behavior. Note: ns: not significant, * *p* < 0.05.

Immoral behavior. In evaluating immoral behavior, a one-way ANOVA was conducted, with immoral behavior as the dependent variable. The analysis showed a significant main effect across groups (*F*(2, 147) = 4.51, *p* < 0.05, η^2^ = 0.06). A post hoc analysis showed that immoral behavior of the MRG (3.38 ± 1.87, *SE* = 0.24, 95%CI [2.91, 3.85]) is significantly higher than that of the MPG (2.48 ± 1.53, *SE*_3_ = 0.24, 95%CI [2.01, 2.95]), while the immoral behavior of the latter is not significantly different from that of the MAG (2.54 ± 1.61, *SE*_2_ = 0.24, 95%CI [2.07, 3.01]). These findings are summarized in Table 4 and Figure 5 and Figure 6.

### 5.3. Discussion

Our study reaffirmed the findings by Rai and Holyoak [16], highlighting that compared to priming moral absolutism, priming moral relativism tends to foster tolerance and a rise in immoral behavior. Significantly, priming moral pragmatism fosters tolerance while no escalation in immoral behaviors is present. These findings bolster our hypothesis, such that when individuals view moral beliefs as pragmatic tools to address specific problems, they are more likely to accommodate differing viewpoints without compromising their own moral convictions. It appears that while solutions to concrete problems can be accommodated within one’s belief system, a universal moral belief often presents a challenge to such integration. In addition, Study 3 repeated the findings of Study 1 and Study 2, with a more balanced representation of different narratives, thus ruling out the possibility that the results of previous studies were due to a mismatch of materials presented.

## 6. General Discussion

Many moral psychologists have traditionally posited that people inherently perceive their moral beliefs as sacred and universal, responding with outrage when these beliefs are challenged. Our four studies, however, suggest a more nuanced perspective. Studies 1–3 indicate that individuals adopt the stance of “moral believers” primarily when dealing with morality in an abstract form. In contrast, when morality is framed in the context of concrete, practical problems, especially when these problems cannot be solved by old experiences, it is perceived as less universal, and violations elicit reduced moral outrage and social exclusion. Additionally, previous research has indicated that exposure to shallow multicultural experiences often leads individuals toward moral relativism, potentially increasing their propensity for immoral behavior. Our last study, however, revealed that introducing the concept of moral pragmatism, which views moral beliefs as culturally developed solutions to specific challenges, fosters greater tolerance without escalating immoral behaviors. This implies that individuals can maintain both tolerance and moral integrity by conceptualizing morality as a pragmatic approach to specific issues rather than abstract, inviolable values. In summary, our research challenges the notion that people are innately moral believers. We demonstrate that individuals can adopt the role of moral problem-solvers engaging with moral issues effectively when approached in an appropriate manner.

Our findings may shed new light on current debates in moral psychology. A lot of contemporary moral psychology studies focus on moral beliefs, and most studies focus on conflicts between moral beliefs, such as studies on moral dilemmas [35]. Such studies have been fruitful, but they might have only touched one aspect of human moral psychology. Few studies have discussed how people deal with complex and fluctuating real-world problems, especially when these problems are novel and require innovation and creativity to be properly handled. We live in a changing world, yet researchers are eager to fit new problems into old frameworks. The pragmatic view presented here might provide a more progressive approach toward moral psychology. Not surprisingly, the pragmatic view is more common in more “applied” fields of psychology, like business ethics [27,32] and educational psychology [20], and we want to bring awareness of this perspective to our fellow social psychologists.

Our findings also have several implications for social psychology in general. Firstly, the debate of whether people are bound by their inherent nature or are shaped by the situation is as old as social psychology itself [36]. Our findings suggest that the answer to this debate is perhaps bound by the situation, as well. People can be dogmatic and stereotypical when moral issues are discussed in the abstract, yet they can also be flexible when a concrete problem must be solved. This phenomenon may not be limited to moral issues but reflects a general tendency for all values, beliefs, and ideas, as suggested by early functionalist psychologists [18,21,22]. This more general hypothesis, along with a more nuanced definition of what makes a situation “abstract” or “concrete”, still needs to be tested in future studies.

Furthermore, our research injects optimism into the field of cross-cultural interactions. Traditional views on multiculturalism often portray foreign values as threats to one’s own framework, leading to either outright rejection or self-doubt [5]. In addition, people often show a negative attitude toward culture mixing and want to keep their own culture “pure” [37]. Our findings, however, suggest that when cultural values and beliefs are approached as practical solutions to specific cultural or societal challenges, exposure to multiculturalism can foster tolerance without undermining confidence in one’s own convictions. This approach recognizes the diversity of problems faced by different cultures and their corresponding unique solutions. Understanding that there is no one-size-fits-all solution allows for the coexistence of multiple belief systems without diminishing one’s belief in their own values. Beyond minimal tolerance, throughout history, different cultures have often learned from each other. Not only are new technologies adopted from foreign cultures but also new thoughts, beliefs, and values. For example, the modern civil service system was first developed in China but was widely adopted by Western countries during the late 19th century. Similarly, foreign religions like Buddhism and Christianity spread to China and have been integrated into local lives. Whether a pragmatic perspective could foster active learning from other cultures is a topic that needs further studies.

All our participants came from China, which might raise the concern of cultural differences. For example, one might argue that under a collectivist culture, Chinese people might be inclined to support government decisions, whatever they are. However, our Chinese participants had diverse views on the social issues we presented, and the extent to which they supported the government varied across different policies. In addition, the materials we used involved stories that happened in unfamiliar foreign countries, so it is unlikely that they received unconditional support from our Chinese participants. Furthermore, when moral issues were presented as abstract and general moral beliefs, our Chinese participants behaved very similarly to Western participants in previous studies. They were also intolerant when moral issues were presented in the abstract. As a result, there are reasons to believe that our findings are not due to something unique to Chinese culture.

Another issue worth mentioning is that in Study 1 and Study 3, we excluded participants who disagreed with the moral belief or policy in the analysis because those who disagreed with certain moral beliefs or policies would not consider them universal. Since participants who agreed with certain moral beliefs or policies varied across different scenarios, there is no need to worry about systematic bias. In our studies, we did not have enough participants to conduct a systematic comparison between those who agreed and those who disagreed. Future studies may use controversial social issues on which people’s attitudes are more evenly distributed to further investigate this issue. To avoid the loss of participants, future studies may also ask participants to come up with moral beliefs that they think are universal, as previous studies on moral beliefs did [13,14], and then make them think about real-world practical challenges.

Although our focus was on universalism in Study 3, we also had some interesting results on attitudinal change. Whether moral beliefs were presented as an idle claim or a decision in a complex situation, participants in Study 3 made similar moral and universal judgments. However, they also reported more attitudinal change when challenging situations were presented. This might indicate a deeper understanding of the issue involved. After considering the complex situation, participants might turn from simple and thoughtless support to nuanced and well-informed support. Future studies could investigate if such a deepening of understanding has further implications, like tolerance of different opinions or willingness to engage in discussions with others.

The current research also has some limitations. Firstly, extreme scenarios were employed to highlight the contrast between abstract beliefs and specific problems. This methodological choice, while effective for this study’s purposes, may not accurately reflect the complexities of real-life situations in which previous beliefs often contribute to problem-solving and contradictions are less pronounced. In more nuanced and complicated situations, individual differences could also play a bigger role. The observed discrepancy between existing beliefs and emergent problems suggests potential opportunities for updating one’s knowledge and adapting to new circumstances. However, previous studies have found that individuals can exhibit dogmatism, resisting changes even in the face of new information [38]. Future research is required to explore what makes individuals adapt their moral understanding in response to new situations and what makes them stick to their old beliefs even when faced with failure. Furthermore, people differ in the degree to which they endorse certain moral beliefs. Future studies could investigate whether stronger believers are less likely to adapt to new situations.

Secondly, our exploration was confined to a limited set of moral beliefs. While we focused on widely recognized beliefs such as monogamy, this does not encompass the entire spectrum of moral convictions that individuals might hold sacred [13,14]. Given the diversity of moral domains identified in previous studies [2], further research is needed to examine whether similar patterns of moral problem-solving apply across a broader range of moral beliefs. In addition, pragmatists like Dewey [21,22] traditionally made no distinction between moral experiences and other experiences like aesthetic experiences. Further studies are needed to investigate if the same pattern occurs in non-moral beliefs. Furthermore, according to moral pragmatists like Dewey [21,22], moral beliefs themselves are the result of experience. When the same solution works repeatedly, it becomes automatic, thus forming an abstract, decontextualized belief. Our current research focuses on the effect of conditions, and more complex and more nuanced designs are needed to capture the transformation, which is beyond the scope of the current paper.

Thirdly, our studies are based on online surveys, and the scenarios we used mostly happened in foreign countries. As a result, participants might feel detached. Future studies could investigate if the same results can be found with controversial domestic issues. In addition, following previous research [11,13], we used national policies as our experimental material. Whether the same effect can be found with more personal issues, like the euthanasia of a relative, remains to be investigated by future studies. Along the same line, in the last study, we used minor online transgression as a measure of immoral behavior, and although the same measure has been used in past studies [16], it may lack ecological validity. Future studies could investigate if the priming effect is strong enough to influence more serious immoral behaviors in real life.

Finally, the underlying mechanism differentiating moral believers from moral problem-solvers warrants further investigation. Our perspective is that moral beliefs represent crystallized experiences, leading individuals to rely on past solutions rather than considering new approaches. This inertia may stem from a tendency to avoid the cognitive effort of reassessing established beliefs. From our perspective, situational pressure trumps individual differences. Alternatively, the divergence in responses to abstract beliefs versus specific problems could be attributed to distinct cognitive processes, paralleling the varied thinking styles found in different cultural contexts [39]. Future studies should explore whether these represent two generalized cognitive approaches to moral reasoning, deepening our understanding of how people navigate moral dilemmas.

## 7. Conclusions

Many moral psychologists have traditionally portrayed individuals as steadfast moral believers, often suggesting an inevitable clash of differing moral beliefs. Our research endeavors, encompassing four distinct studies, offer an alternative approach. These studies collectively suggest that when moral questions are framed in a context-specific and pragmatic manner, individuals can adopt the role of adaptable and inclusive moral problem-solvers. This alternative approach underscores the potential for more harmonious and flexible engagements in the face of diverse moral viewpoints, thereby challenging the traditional narrative of inevitable moral conflict.

## Figures and Tables

**Table 1:** Comparative analysis of moral judgment and universalism scores across scenarios for the abstract value group (AVG) and specific problem group (SPG).

Scenario	Variables	Group	*M*	*SD*	*df*	*t*	*p*-*Value*	Cohen’s *d*
Birth Control	Judgment	AVG	5.94	1.10	152	2.17	<0.05 *	0.35
		SPG	5.60	0.84				
	Universal	AVG	5.06	1.36	152	3.90	<0.01 **	0.66
		SPG	4.00	1.82				
Polygamy	Judgment	AVG	6.27	0.91	136	6.07	<0.001 ***	1.08
		SPG	5.24	1.00				
	Universal	AVG	5.20	1.49	136	5.78	<0.001 ***	1.02
		SPG	3.57	1.70				
Land Reform	Judgment	AVG	5.77	0.86	173	0.55	0.58	0.09
		SPG	5.84	0.78				
	Universal	AVG	4.79	1.57	173	2.95	<0.01 **	0.45
		SPG	4.05	1.71				

Note: * *p* < 0.05, ** *p* < 0.01, *** *p* < 0.001.

**Table 2:** Comparative analysis of moral judgment, outrage, and tolerance among groups endorsing three moral perspectives, including agree with abstract value (AAV), agree with specific solution (ASS), and disagree with specific solution (DSS).

Variables	Group	*M*	*SD*	*df*	*F*	*p*-Value	η^2^
Judgment	AAV	6.28	0.88	193	483.21	<0.001 ***	0.83
	ASS	5.56	0.92				
	DSS	2.15	0.74				
Outrage	AAV	5.35	1.36	193	19.93	<0.001 ***	0.17
	ASS	3.59	1.71				
	DSS	5.00	1.34				
Tolerance	AAV	2.43	1.18	193	18.42	<0.001 ***	0.16
	ASS	3.72	1.43				
	DSS	2.34	1.01				

Note: *** *p* < 0.001.

**Table 3:** Comparative analysis of moral judgment and universalism scores across scenarios for abstract value group (AVG), value consistent group (VCG), and specific problem group (SPG).

Scenario	Variables	Group	*M*	*SD*	*df*	*F*	*p*-*Value*	η^2^
Birth Control	Judgment	AVG	6.32	0.67	192	12.10	<0.001 ***	0.11
		VCG	5.71	0.86				
		SPG	5.80	0.79				
	Universal	AVG	5.20	1.51	192	15.67	<0.001 ***	0.14
		VCG	4.76	1.45				
		SPG	3.76	1.90				
	Attitude Change	AVG	2.81	1.74	192	26.46	<0.001 ***	0.22
		VCG	4.52	1.40				
		SPG	4.60	1.67				
Polygamy	Judgment	AVG	6.29	1.10	218	24.08	<0.001 ***	0.18
		VCG	6.16	0.69				
		SPG	4.97	1.19				
	Universal	AVG	5.13	1.43	218	35.50	<0.001 ***	0.25
		VCG	5.30	1.31				
		SPG	2.93	1.81				
	Attitude Change	AVG	2.49	1.61	218	27.86	<0.001 ***	0.20
		VCG	3.60	2.12				
		SPG	5.09	1.16				
Land Reform	Judgment	AVG	5.97	0.78	199	4.43	<0.05 *	0.04
		VCG	5.61	0.78				
		SPG	6.07	0.69				
	Universal	AVG	4.86	1.46	199	2.28	=0.10	0.02
		VCG	4.31	1.69				
		SPG	4.37	1.72				
	Attitude Change	AVG	3.04	1.57	199	24.68	<0.001 ***	0.20
		VCG	4.26	1.93				
		SPG	4.79	1.56				

Note: * *p* < 0.05, *** *p* < 0.001.

**Table 4:** Comparative analysis of persuasiveness, attitude change, tolerance, and immoral behavior across different moral perspectives, including moral relativism group (MRG), moral pragmatism group (MPG), and moral absolutism group (MAG).

Variables	Group	*M*	*SD*	*df*	*F*	*p*-Value	η^2^
Persuasiveness	MRG	4.64	1.76	147	5.66	<0.01 **	0.08
	MPG	5.10	1.49				
	MAG	5.68	1.38				
Attitude Change	MRG	3.60	1.74	147	0.45	0.64	0.01
	MPG	3.32	1.73				
	MAG	3.64	2.02				
Tolerance	MRG	3.96	1.99	147	13.21	<0.001 ***	0.15
	MPG	3.10	1.87				
	MAG	2.16	1.33				
Immoral Behavior	MRG	3.38	1.87	147	4.51	<0.05 *	0.06
	MPG	2.48	1.53				
	MAG	2.54	1.61				

Note: * *p* < 0.05, ** *p* < 0.01, *** *p* < 0.001.

## Data Availability

The data presented in this study are available upon request via email to the corresponding author. Please indicate the purpose of the research and include the statement of data confidentiality in the email.

## Supplementary Materials

The following supporting information can be downloaded at: https://www.mdpi.com/article/10.3390/bs14110984/s1.

## Appendix A

Experimental Materials

Studies 1 and 2

Birth Control

Abstract value: The number of children to have is a right of women, and the government does not have the right to intervene.

Specific solution: Bangladesh is a poor country located in South Asia. In Bangladesh, a family will have four to five children on average. In recent years, as the economic and sanitary situation has improved, Bangladesh has experienced a population explosion. At the same time, Bangladesh cannot provide enough jobs for the increased population due to the pandemic. This leads to a lot of jobless young people wandering around in the cities, and the food supply is becoming tighter and tighter. Bangladeshi society is facing significant pressure regarding jobs, ecology, and food. To deal with this situation, the Bangladeshi government plans to implement birth control, allowing each woman to have at most two children.

Polygamy

Abstract value: Monogamy is the only legitimate marital institution. Polygamy is unacceptable.

Specific solution: The Chad Republic is a poor developing country in Middle Africa. Chad’s political situation is unstable. It has had several civil wars in the past decade, causing many deaths of young men. Chad’s situation stabilized recently, and the new government found that the gender ratio is severely out of balance due to war. The ratio of men to women is 1:2. This situation has affected the stability of Chad’s society and challenged its traditional marital values. Traditionally, Chad has had monogamy, which was also confirmed by the law. However, faced with a dire situation, Chad’s government has decided to change the law and implement polygamy, allowing a man to have two wives.

Land Reform

Abstract value: The government must protect private property and should not conduct wealth redistribution that takes from the few and gives to the many.

Specific solution: South Africa is a relatively rich country in Africa. However, South African society is extremely unequal, with 76% of the land owned by 1% of the population. The vast majority of the people either live in slums in big cities or rent land from big landlords. With the shock of the pandemic, this twisted social structure caused unsettlement among the people and led to social upheaval. To deal with the problem, the new South African government plans to buy back land from the big landlords at a low price and then distribute the land to the poor peasants.

Study 3

Birth Control

Value consistency: Bangladesh is a poor country located in South Asia. In Bangladesh, a family will have four to five children on average. In recent years, as the economic and sanitary situation has improved, Bangladesh has experienced a population explosion. At the same time, Bangladesh cannot provide enough jobs for the increased population due to the pandemic. This has led to a lot of jobless young people wandering around in the cities, and the food supply is becoming tighter and tighter. Bangladeshi society is facing significant pressure regarding jobs, ecology, and food. Faced with such a situation, the Bangladeshi government refuses to interfere because they believe that the number of children to have is a right of women, and the government does not have the right to intervene.

Polygamy

Value consistency: The Chad Republic is a poor developing country in Middle Africa. Chad’s political situation is unstable. It has had several civil wars in the past decade, causing many deaths of young men. Chad’s situation has stabilized recently, and the new government has found that the gender ratio is severely out of balance due to war. The ratio of men to women is 1:2. This situation has affected the stability of Chad’s society and challenged its traditional marital values. Traditionally, Chad has had monogamy, which was also confirmed by the law. Faced with a dire situation, Chad’s government has decided to keep the old marital policy because they believe that monogamy is the only legitimate marital institution. Polygamy is unacceptable.

Land Reform

Value consistency: South Africa is a relatively rich country in Africa. However, South African society is extremely unequal, with 76% of the land owned by 1% of the population. The vast majority of the people either live in slums in big cities or rent land from big landlords. With the shock of the pandemic, this twisted social structure has caused unsettlement among the people and led to social upheaval. Faced with this problem, the new South African government has decided not to redistribute the land because they believe that the government must protect private property and should not conduct wealth redistribution that takes from the few and gives to the many.

Study 4

Moral absolutism

Morality concerns principles about right and wrong. These principles have absolute boundaries and should be followed by anyone under any circumstances.

Take marriage as an example. Humanity has had many different marital institutions throughout history. Islam allows men to have four wives; ancient China permitted men to have concubines beyond their wives; the Medieval Catholic church did not allow remarriage after divorce; ethnic minorities in Southern China have “walking marriages”, in which each night, men sleep at women’s houses and leave children for the women’s families; and in Cameroon, after the father dies, the son must marry his father’s wives, except for his own mom. These institutions objectify and discriminate against women, treating women as mere tools for reproduction or property of men, not as equal and independent of men. As a result, these marital institutions are immoral. Only modern monogamy recognizes the equality of men and women; thus, it is the marital institution that fits with moral principles.

As a result, we should not tolerate marital institutions of other cultures because they are immoral. Our moral beliefs are objective knowledge of right and wrong. They are universal and should be followed unconditionally.

Moral relativism

Morality is shaped by culture and growth. There is no absolute boundary to morality, and what is right or wrong depends on the specific situation.

Take marriage as an example. Humanity has had many different marital institutions throughout history. Islam allows men to have four wives; ancient China permitted men to have concubines beyond their wives; the Medieval Catholic church did not allow remarriage after divorce; ethnic minorities in Southern China have “walking marriages”, in which each night, men sleep at women’s houses and leave children for the women’s families; and in Cameroon, after the father dies, the son must marry his father’s wives, except for his own mom. For each culture, their own marital institution is the most reasonable and moral. Monogamy is only one custom among many, and it is also the product of our culture.

As a result, we cannot simply make judgments of other cultures or put our moral values onto them. If we were born into a culture of polygamy, we would consider it right. We must recognize that our moral beliefs are mere products of our culture, rather than some objective standard.

Moral pragmatism

Morality is a cultural phenomenon that human society evolves to deal with specific problems. Although different cultures have different moral values, they are all results of adaptation to the environment.

Take marriage as an example. Humanity has had many different marital institutions throughout history. Islam allows men to have four wives because war once caused a sharp decline in the male population; ancient China relied on manpower to plow the land, so it permitted men to have concubines beyond their wives to secure a male offspring; ethnic minorities in Southern China live on foraging performed by women, so it has “walking marriages”, in which each night, men sleep at women’s houses and leave children for the women’s families; and in Cameroon, after the father dies, the son must marry his father’s wives, except for his own mom, to make sure that family property will not be lost. Monogamy in modern society is also a product of industrialization.

As a result, we cannot simply make judgments of other cultures or put our moral values onto them. We must proceed case by case and investigate the specific situation and practical problem a culture faces, and judgment should be made on that basis, as well.
