# Supplementary material for: Moral Believer or Moral Problem-Solver? Moral Pragmatism Fosters Tolerance Without Impeding Moral Behavior
Source: Behav Sci (Basel). 2024 Oct 23;14(11):984. doi: 10.3390/bs14110984 (PMC11590898; doi:10.3390/bs14110984)
Supplement: Supplementary file 1 [file behavsci-14-00984-s001.zip › behavsci-3212626-Supplementary Materials.pdf]

Experimental Materials

Study 1 & 2		
Birth Control	**Abstract value**: To have how many children is a right of women, and the government has not right to intervene.	**抽象道德信念**：生育多少孩子是女性自己的权利，政府无权干涉
	**Specific solution**: Bangladesh is a poor country located in South Asia. In Bangladesh, a family will have four to five children on average. In recent years, as economic and sanitary situation improves, Bangladesh experiences an explosion of population. At the same time, Bangladesh cannot provide enough jobs for the increased population due to the pandemic. This leads to a lot of jobless young people wandering around in the cities and the food supply is becoming tighter and tighter. The Bangladesh society is facing huge pressure on jobs, ecology and food. To deal with such situation, Bangladesh government plans to implement birth control, allowing each woman to have at most two children.	**具体问题解决**：孟加拉位于南亚，是一个贫穷的农业国家。在孟加拉，平均一户人家通常会生育 4-5 个孩子。近年来，随着经济发展和卫生条件的提高，孟加拉的人口经历了爆炸性的增长。同时，受疫情影响，孟加拉无法发展工业来为新增人口提供就业。这一情况在农村造成人多地少，在城市则产生了大量待业青年，这使得粮食供给越来越紧张，社会面临巨大的就业压力、生态压力和粮食压力。为了应对这一局面，孟加拉政府计划推行计划生育政策，规定每名妇女一生最多只能生育两个孩子。
Polygamy	**Abstract value**: Monogamy is the only legitimate marital institution. Polygamy is unacceptable.	**抽象道德信念**：在现代社会，一夫一妻制是唯一合理的制度，一夫多妻制是不可接受的
	**Specific solution**: The Chad Republic is a poor developing country in Middle Africa. Chad's political situation is unstable. It had several civil wars in the past decade, causing lots of death of young men. Chad's situation stabilized recently, and the new government found that gender ratio is severely out of balance due to war. The ratio of number of men to women is 1: 2. This situation affected the stability of Chad society and challenged its traditional marital values. Traditionally Chad had monogamy, which was also confirmed by law. However, faced with the dire situation, Chad government decides to change the law and implement polygamy, allowing a man to have two wives.	**具体问题解决**：乍得共和国位于非洲中部，是一个贫穷的发展中国家。乍得的政治局势并不稳定，在几年时间内经历了好几轮内战，造成大量青年男性死亡。直到最近乍得的局势才稳定下来，此时新政府发现由于连年战乱乍得国内的性别比例严重失衡，男女比例达到了惊人的 1：2。这一情况影响了乍得的社会稳定，也对传统的婚姻价值观造成了挑战。传统上乍得一直都实行一夫一妻制度，而这一制度也以法律的形式被确定下来。然而，面对严峻的现实情况，乍得政府决定修改法律，实行一夫多妻制，允许男性娶一到两个妻子。
Land Reform	**Abstract value**: The government must protect private property and should not conduct wealth redistribution that take from the few and give to the many.	**抽象道德信念**：政府必须保护私有财产，不能搞劫富济贫式的财富再分配
	**Specific solution**: South Africa is a relatively rich country in Africa. However, the South African society is	**具体问题解决**：南非共和国位于非洲最南端，是一个在非洲相对较为富裕的国

	extremely unequal, with 76% of the land owned by 1% of the population. The vast majority of the people either live in slums in big cities or rent land from big landlords. With the shock of the pandemic, this twisted social structure caused unsettlement among the people and led to social upheaval. To deal with the problem, the new South African government plans to buy back land from the big landlord at a low price, and then distribute the land to the poor peasants.	家。然而，南非社会的财富分布极度不平等，全国 1%的人口拥有全国 76%的土地，而全国绝大部分人口要么居住在城市的贫民窟中，要么不得不向大地主借贷，租用大地主的土地耕种。在新冠疫情的冲击下，这种畸形的社会结构激发了底层人民的不满，引起了社会动荡。为了应对这一问题，新一届的南非政府计划以较低的价格征收和赎买大地主手中的土地，并将土地分给无地的贫民。
**Study 3**		
Birth Control	**Value consistency**: Bangladesh is a poor country located in South Asia. In Bangladesh, a family will have four to five children on average. In recent years, as economic and sanitary situation improves, Bangladesh experiences an explosion of population. At the same time, Bangladesh cannot provide enough jobs for the increased population due to the pandemic. This leads to a lot of jobless young people wandering around in the cities and the food supply is becoming tighter and tighter. The Bangladesh society is facing huge pressure on jobs, ecology and food. **Faced with such situation, Bangladesh government refuses to interfere, because they believe that to have how many children is a right of women, and the government has not right to intervene.**	**信念一致决策**：孟加拉位于南亚，是一个贫穷的农业国家。在孟加拉，平均一户人家通常会生育 4-5 个孩子。近年来，随着经济发展和卫生条件的提高，孟加拉的人口经历了爆炸性的增长。同时，受疫情影响，孟加拉无法发展工业来为新增人口提供就业。这一情况在农村造成人多地少，在城市则产生了大量待业青年，这使得粮食供给越来越紧张，社会面临巨大的就业压力、生态压力和粮食压力。**面对这一局面，孟加拉政府决定继续施行不干预生育的政策，因为他们相信生育多少孩子是女性自己的权利，政府无权干涉。**
Polygamy	**Value consistency**: The Chad Republic is a poor developing country in Middle Africa. Chad's political situation is unstable. It had several civil wars in the past decade, causing lots of death of young men. Chad's situation stabilized recently, and the new government found that gender ratio is severely out of balance due to war. The ratio of number of men to women is 1: 2. This situation affected the stability of Chad society and challenged its traditional marital values. Traditionally Chad had monogamy, which was also confirmed by law. **Faced with the dire situation, Chad government decides to keep the old marital policy, because they believe that monogamy is the only legitimate marital institution. Polygamy is unacceptable.**	**信念一致决策**：乍得共和国位于非洲中部，是一个贫穷的发展中国家。乍得的政治局势并不稳定，在几年时间内经历了好几轮内战，造成大量青年男性死亡。直到最近乍得的局势才稳定下来，此时新政府发现由于连年战乱乍得国内的性别比例严重失衡，男女比例达到了惊人的 1：2。这一情况影响了乍得的社会稳定，也对传统的婚姻价值观造成了挑战。传统上乍得一直都实行一夫一妻制度，而这一制度也以法律的形式被确定下来。**面对严峻的现实情况，乍得政府决定继续维持过去的婚姻政策，因为他们相信一夫一妻制是唯一合理的制度，一夫多妻制是不可接受的。**

Land Reform	**Value consistency**: South Africa is a relatively rich country in Africa. However, the South African society is extremely unequal, with 76% of the land owned by 1% of the population. The vast majority of the people either live in slums in big cities or rent land from big landlords. With the shock of the pandemic, this twisted social structure caused unsettlement among the people and led to social upheaval. **Faced with the problem, the new South African government decides not to redistribute the land, because they believe that government must protect private property and should not conduct wealth redistribution that take from the few and give to the many.**	**信念一致决策：**南非共和国位于非洲最南端，是一个在非洲相对较为富裕的国家。然而，南非社会的财富分布极度不平等，全国 1%的人口拥有全国 76%的土地，而全国绝大部分人口要么居住在城市的贫民窟中，要么不得不向大地主借贷，租用大地主的土地耕种。在新冠疫情的冲击下，这种畸形的社会结构激发了底层人民的不满，引起了社会动荡。**面对这一问题，新一届的南非政府决定不征收大地主的土地来搞土地再分配，因为他们相信政府必须保护私有财产，不能搞劫富济贫式的财富再分配。**
**Study 4**		
Moral absolutism	Morality concerns principles about right and wrong. These principles have absolute boundaries and should be followed by anyone under any circumstances.<br><br>Take marriage as an example. Humanity has many different marital institutions throughout history. Islam allows men to have four wives; ancient China permits men to have concubines beyond wife; Medieval Catholic Church did not allow remarriage after divorce; ethnic minorities in Sothern China have “walking marriage”, with which each night men sleep at women's house and leave children to women's family; in Cameroon, after the father dies the son must marry his father's wives except his own mom. These institutions objectify and discriminates against women, treating women as mere tools for reproduction or property of men, not as equal and independent of men. As a result, these marital institutions are immoral. Only the modern monogamy recognizes the equality of men and women, thus it is the marital institution that fits with moral principles.<br><br>As a result, we should not tolerate marital institutions of other cultures, because they are immoral. Our moral beliefs are objective knowledge of right and wrong. They are universal and should be followed unconditionally.	道德是关于是非对错的原则。这些原则有着绝对清晰的界限，任何人在任何情况下都应该遵守它们。<br>以婚姻制度为例，人类在不同历史时期不同文化中有着极为不同的婚姻制度。伊斯兰教允许男性娶四个妻子；中国古代实行一夫一妻多妾制，男性在正妻之外还可以有小妾；中世纪天主教不允许女性在离婚后改嫁；我国南方的少数民族摩梭人实行走婚制，每天晚上成年男人到自己意中人的家中幽会，小孩由女方家庭抚养；在喀麦隆，儿子在父亲去世后要娶除自己亲生母亲外父亲的其他妻子。这些制度往往是歧视和物化女性的产物，它们仅仅把女性当作生育的工具或是从属于男性的私有财产，而非具有独立人格，且与男性平等的人。因此，这些婚姻制度都是不道德的。只有现代的一夫一妻制才真正实现了男女平等，因而也是最符合道德原则的婚姻制度。<br>因此，对于婚姻制度我们不应该容忍其他文化的不同制度，因为它们是不道德的。我们的道德信念是对于是非对错的客观认识，它们是普遍成立的，而我们在任何情况下都应该遵守我们的道德原则。

Moral relativism	Morality is shaped by culture and growth. There is no absolute boundary to morality, and what is right or wrong depends on the specific situation.<br><br>Take marriage as an example. Humanity has many different marital institutions throughout history. Islam allows men to have four wives; ancient China permits men to have concubines beyond wife; Medieval Catholic Church did not allow remarriage after divorce; ethnic minorities in Sothern China have "walking marriage", with which each night men sleep at women's house and leave children to women's family; in Cameroon, after the father dies the son must marry his father's wives except his own mom. For each culture, their own marital institution is most reasonable and moral. Monogamy is only one custom among many, and it is also the product of our culture.<br><br>As a result, we cannot simply make judgments on other cultures or put our moral values onto them. If we were born in a culture with polygamy, we would consider it right. We must recognize that our moral beliefs are mere products of our culture, rather than some objective standard.	道德是由文化和成长经历塑造的。道德没有绝对清晰的界限，什么是对什么是错完全取决于具体情况。<br>以婚姻制度为例，人类在不同历史时期不同文化中有着极为不同的婚姻制度。伊斯兰教允许男性娶四个妻子，但娶新的妻子需要得到已有妻子的同意；中国古代实行一夫一妻多妾制，男性在正妻之外还可以有小妾；我国南方的少数民族摩梭人实行走婚制，他们没有固定的婚姻关系，每天晚上成年男人到自己意中人的家中幽会，到第二天早上又回到自己的家中，小孩则由女方家庭抚养；在喀麦隆，儿子在父亲去世后要娶除自己亲生母亲外父亲的其他妻子。在每一个文化看来，自身的婚姻制度都是最合理和符合道德原则的，而现代社会常见的一夫一妻制只不过是多种婚姻制度中的一种，同样也只是我们自身文化的产物。<br>因此，我们不能简单的评价其他文化的道德观，或是把我们的道德价值观强加给他人。如果我们生在一个实行一夫多妻的时代或文化，我们也会觉得这就是正确的。我们必须认识到我们自己的道德信念仅仅是自身文化的产物，而非某种客观的标准。
Moral pragmatism	Morality is a culture phenomenon that human society evolves to deal with specific problems. Although different cultures have different moral values, they are all results of adaptation to the environment.<br><br>Take marriage as an example. Humanity has many different marital institutions throughout history. Islam allows men to have four wives because war once caused a sharp decline in male population; ancient China relies on manpower to plow the land, so it permits men to have concubines beyond wife to secure a male offspring; ethnic minorities in Sothern China lives on foraging by women, so it have "walking marriage", with which each night men sleep at women's house and leave children to women's family; in Cameroon, after the father dies the son must marry his father's wives except his	道德是人类社会为了解决具体问题而演化出的文化现象。虽然各个时期各个文化的道德观不同，但它们都是适应当时环境和生产力的产物。<br>以婚姻制度为例，人类在不同历史时期不同文化中有着极为不同的婚姻制度。中东地区曾因战乱导致男性人口大幅减少，因而伊斯兰教允许男性娶四个妻子；中国古代是小农社会，男性劳动力对于生存极为重要，所以允许娶妾以保证男性后代的数量；我国南方的少数民族摩梭人主要依靠女性采集野果和捕鱼为生，不依靠男性劳动力，因此实行走婚制，每天晚上成年男人到自己意中人的家中幽会，小孩由女方家庭抚养；

	own mom to make sure that family property will not leave. Monogamy in modern society is also a product of industrialization.<br><br>As a result, we cannot simply make judgments on other cultures or put our moral values onto them. We must go case by case and investigate the specific situation and practical problem a culture faces, and judgment should be made on that basis as well.	在喀麦隆，儿子在父亲去世后要娶除自己亲生母亲外父亲的其他妻子，以保证不会因改嫁发生家族财产的分裂；而现代社会常见的一夫一妻制也是为了适应工业化生产方式而产生的。<br>因此，我们不能简单的评价其他文化的道德观，或是把我们的道德价值观强加给他人。我们必须具体问题具体分析，考察该文化的具体情况和面对的实际问题，在此基础上才能对其他文化的道德价值观做出我们的评判。
